# Evaluation of the influenza sentinel surveillance system in the Democratic Republic of Congo, 2012–2015

**DOI:** 10.1186/s12889-019-8008-2

**Published:** 2019-12-10

**Authors:** Pélagie Babakazo, Joelle Kabamba-Tshilobo, Emile Okitolonda Wemakoy, Léopold Lubula, Léonie Kitoko Manya, Benoit Kebela Ilunga, Wally Disasuani, Edith Nkwembe, Hugo Kavunga-Membo, Jean-Claude Changachanga, Saleh Muhemedi, Jean-Jacques Muyembe Tamfum, Stefano Tempia

**Affiliations:** 1 Kinshasa School of Public Health, University of Kinshasa, Kinshasa, Democratic Republic of Congo; 2 Influenza and Monkeypox Program, Centers for Disease Control and Prevention, Kinshasa, Democratic Republic of Congo; 3 Division de Lutte Contre la Maladie, Ministry of Health, Kinshasa, Democratic Republic of Congo; 4 Institut National de Recherche Biomédicale, Ministry of Health, Kinshasa, Democratic Republic of Congo; 5 Influenza Division, Centers for Disease Control and Prevention, Atlanta, GA USA; 6 Influenza Program, Centers for Disease Control and Prevention, Pretoria, South Africa; 7 MassGenics, Duluth, GA USA; 8 Center for Respiratory Diseases and Meningitis, National Institute for Communicable Diseases, Private Bag X4, Sandringham, Gauteng, 2131 South Africa

**Keywords:** Influenza, Surveillance, Evaluation, Democratic Republic of Congo

## Abstract

**Background:**

The World Health Organization recommends periodic evaluations of influenza surveillance systems to identify areas for improvement and provide evidence of data reliability for policymaking. However, data about the performance of established influenza surveillance systems are limited in Africa, including in the Democratic Republic of Congo (DRC).

**Methods:**

We used the Centers for Disease Control and Prevention guidelines to evaluate the performance of the influenza sentinel surveillance system (ISSS) in DRC during 2012–2015. The performance of the system was evaluated using eight surveillance attributes: (i) data quality and completeness for key variables, (ii) timeliness, (iii) representativeness, (iv) flexibility, (v) simplicity, (vi) acceptability, (vii) stability and (viii) utility. For each attribute, specific indicators were developed and described using quantitative and qualitative methods. Scores for each indicator were as follows: < 60% weak performance; 60–79% moderate performance; ≥80% good performance.

**Results:**

During 2012–2015, we enrolled and tested 4339 patients with influenza-like illness (ILI) and 2869 patients with severe acute respiratory illness (SARI) from 11 sentinel sites situated in 5 of 11 provinces. Influenza viruses were detected in 446 (10.3%) samples from patients with ILI and in 151 (5.5%) samples from patients with SARI with higher detection during December–May. Data quality and completeness was > 90% for all evaluated indicators. Other strengths of the system were timeliness, simplicity, stability and utility that scored > 70% each. Representativeness, flexibility and acceptability had moderate performance. It was reported that the ISSS contributed to: (i) a better understanding of the epidemiology, circulating patterns and proportional contribution of influenza virus among patients with ILI or SARI; (ii) acquisition of new key competences related to influenza surveillance and diagnosis; and (iii) continuous education of surveillance staff and clinicians at sentinel sites about influenza. However, due to limited resources no actions were undertaken to mitigate the impact of seasonal influenza epidemics.

**Conclusions:**

The system performed overall satisfactorily and provided reliable and timely data about influenza circulation in DRC. The simplicity of the system contributed to its stability. A better use of the available data could be made to inform and promote prevention interventions especially among the most vulnerable groups.

## Background

Global influenza surveillance, coordinated by the World Health Organization (WHO) under the Global Influenza Surveillance and Response Network (GISRN), is key to monitoring global trends of seasonal influenza virus circulation, guiding strain selection for annual influenza vaccine composition, monitoring acquisition of resistance to antiviral drugs, detecting the emergence of influenza viruses with pandemic potential, and monitoring the spread and impact of pandemic influenza viruses. WHO recommends the use of standard case definitions and procedures for global influenza surveillance among outpatients and inpatients as well as periodic comprehensive evaluations of established surveillance systems, beginning 1–2 years after implementation [1, 2].

Guidelines from the United States (US) Centers for Disease Control and Prevention (CDC) [3, 4] suggest that *“the usefulness of a surveillance system is dependent on the actions that can be taken as a result of data collection and analysis; specifically, whether the system is able to: (i) guide disease prevention and control activities through the timely detection of adverse health-events, (ii) estimate the magnitude of morbidity and mortality and associated risk factors, (iii) detect trends that signal changes in incidence, including epidemics, (iv) permit assessment of prevention and control measures, (v) lead to improved health and social policy or clinical practice, and (vi) stimulate research to inform prevention and control measures”.*

During the past decade, influenza sentinel surveillance has been established in several African countries [5] including the Democratic Republic of Congo (DRC) [6]. Given the geographic location of the country, which is situated along important bird migratory routes, and the close contact of the population with domestic and wild birds, the influenza sentinel surveillance system in DRC was established with the aim to monitor the circulating seasonal influenza strains as well as to detect emerging zoonotic viruses. No influenza treatment or immunization guidelines are currently available in low-income DRC because of competing priorities with other diseases and limited financial resources. However, data from the established surveillance system, if accurate and reliable, could inform and promote prevention interventions.

Although influenza sentinel surveillance has been established in several African countries, data about the performance of established surveillance systems are limited on the continent [7–10]. Such evaluations would enable countries to assess the performance of their surveillance systems, identify areas for improvement and provide evidence of data reliability for policymaking and public health interventions as well as compliance with international surveillance standards.

We conducted a systematic evaluation of the national influenza surveillance system implemented among outpatients with influenza-like illness (ILI) and inpatients with severe acute respiratory illness (SARI) during January 2012 through December 2015. Findings from this evaluation will help to improve the performance of the influenza surveillance system in DRC.

## Methods

### Overview of the influenza surveillance system during 2012–2015

An influenza sentinel surveillance system (ISSS) was established in DRC in 2006 following the emergence of the highly pathogenic avian influenza A(H5N1) strain in Asia with a high case-fatality rate [11]. The objectives of the DRC-ISSS are to: (i) detect and respond to influenza outbreaks; (ii) assess the proportion of patients meeting the ILI and SARI case definition that is attributable to influenza virus infection; (iii) assess the burden of influenza-associated illness; (iv) monitor the temporal trends of influenza virus circulation; (v) monitor the circulating influenza virus types and subtypes annually; (vi) maintain laboratory capacity for seasonal and avian influenza viruses detection; and (vii) obtain and share clinical samples for annual selection of influenza virus strains for influenza vaccine formulation under the WHO-GISRN. In addition, the data generated through the surveillance system were considered key to potentially inform and promote prevention interventions. The ISSS was coordinated and implemented by three institutions, namely: the Kinshasa School of Public Health (KSPH), the Direction de la Lutte contre les Maladies (DLM) and the Institut National de Recherche Biomédicale (INRB). International stakeholders included the CDC and WHO Country Offices.

Surveillance was designed to be implemented in 6 of 11 provinces of the country. During the review period (2012–2015) the ISSS included 5 of 6 target provinces. Although health facilities were available in all provinces, provinces to be covered were selected based on available financial resources and pre-established criteria. Priority was given to provinces situated at major entry points of the country with significant population density and movement and to those located along the corridors of migratory wild birds. The selected provinces were situated throughout the national territory, with the exception of the northwestern part of the country.

**Fig. 1:**
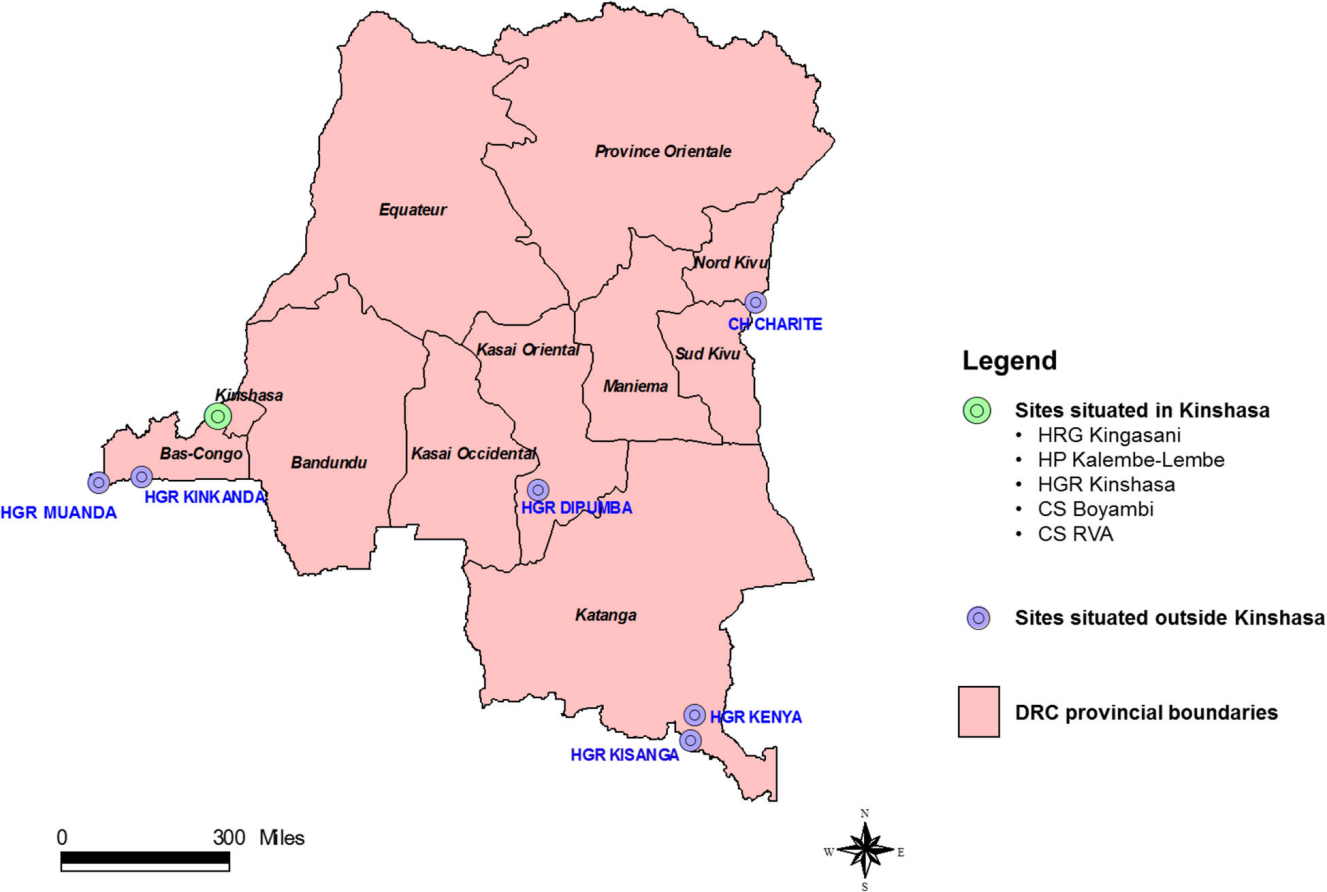
Location of the influenza sentinel surveillance sites in the Democratic Republic of Congo (DRC), 2012–2015

In total 11 sentinel sites (2 clinics implementing ILI surveillance only and 9 hospitals implementing both ILI and SARI surveillance) located in 5 provinces were included in this evaluation (Table 1 and Fig. 1). In the 9 hospitals, ILI surveillance was conducted in the outpatient department; whereas SARI surveillance was conducted in the medical pediatric and adult wards. At each sentinel site trained staff (i.e., doctors, nurses or laboratory technicians) would: (i) screen, identify and enroll patients, (ii) compile individual-level screening and enrollment logbooks for patients meeting the SARI and ILI case definitions, (iii) collect upper respiratory tract (URT) samples from enrolled ILI and SARI cases, (vi) complete the individual case investigation form (CIF) for enrolled ILI and SARI cases, (v) store, package and ship URT samples, (vi) collect weekly and monthly aggregated data on the total number of any, respiratory (including those that met the ILI and SARI case definitions), gastrointestinal and malaria outpatient consultations and hospitalizations; and (vii) liaise with the national influenza surveillance focal points on all matters related to influenza surveillance implemented at the sentinel sites, including requests for surveillance material. No monetary incentive was provided to the influenza focal points. Airtime was provided for communication between sentinel sites and national focal points. Nonetheless, the ISSS in DRC was largely dependent (≥90%) on external funds, especially for sample transportation and testing as well as for supervision activities.

**Table 1:** Healthcare facilities implementing influenza sentinel surveillance in the Democratic Republic of Congo, 2012–2015

Province	City	Sentinel site	Sector	Type of surveillance	Year of inception^a^
Kinshasa	Kinshasa	Clinique de Santé RVA	Private	ILI	2006
Kinshasa	Kinshasa	Clinique de Santé Boyambi	Religious	ILI	2006
Kinshasa	Kinshasa	Centre Hospitalier de Kingasani	Religious	ILI and SARI	2006
Kinshasa	Kinshasa	Hôpital Pédiatrique Kalembe-Lembe	Public	ILI and SARI	2006
Kinshasa	Kinshasa	Hôpital Général de Référence de Kinshasa	Public	ILI and SARI	2006
Bas-Congo	Matadi	Hôpital Général de Référence Kinkanda	Public	ILI and SARI	2011
Bas-Congo	Muanda	Hôpital Général de Référence Muanda	Public	ILI and SARI	2011
Katanga	Lubumbashi	Hôpital Général de Référence Kenya	Public	ILI and SARI	2011
Katanga	Lubumbashi	Hôpital Général de Référence Kisanga	Public	ILI and SARI	2011
Kasaï-Oriental	Mbuji-Mayi	Hôpital Général de Référence Dipumba	Public	ILI and SARI	2012
Nord-Kivu	Goma	Centre Hospitalier Charité Maternelle	Religious	ILI and SARI	2013

Abbreviations: *ILI* influenza-like illness, *SARI* severe acute respiratory illness

^a^ All sites were actively implementing influenza surveillance until December 2015

A case of ILI was defined as an ambulatory patient of any age presenting with a recorded temperature ≥ 38 °C and cough or sore throat of duration of ≤7 days. A case of SARI was defined as a hospitalized person who had illness onset within 7 days of admission and who met age-specific clinical inclusion criteria. A case in children aged 2 days to < 5 years included any hospitalized patient with cough or difficulty breathing and at least one of the following danger signs: unable to drink or breastfeed, lethargic, vomits everything, convulsion, chest indrawing or stridor in a calm child. A case in persons aged ≥5 years included any hospitalized patient with fever (≥38 °C), cough and shortness of breath or difficulty breathing. Identification of ILI cases was implemented at the triage area of the participating sites by a combination of clinical examination for relevant symptoms and patient/caregiver interviews. Identification of SARI cases was implemented at the participating wards by bed-to-bed screening of admitted patients and a combination of clinical examination for relevant symptoms, review of medical records and patient/caregiver interviews.

Aggregated weekly data on the total number of identified SARI and ILI cases and those enrolled were sent weekly by short message service (SMS) to the national influenza focal point; whereas the source data collection forms were sent monthly. For enrolled patients surveillance staff completed case report forms that included demographic, clinical and epidemiological information as well as specimens (nasopharyngeal and oropharyngeal swabs). Specimens were placed in the same vial containing universal transport medium, stored at 4–8 °C and transported to the national influenza laboratory (INRB, Kinshasa, DRC) within 72 h of collection for testing. Specimens were tested for influenza A and B viruses using a real-time reverse transcription polymerase chain reaction assay [6]. Influenza A-positive samples were further subtyped [12]. Non-systematic testing for other respiratory viral pathogens including respiratory syncytial virus, human metapneumovirus, parainfluenza virus types 1–4, rhinovirus, coronavirus OC43, 229E, NL63 and HKUI, and adenovirus was also implemented. Verbal informed consent was obtained from all patients prior to data and specimen collection. For children aged < 15 years, verbal consent was obtained from a parent or legal guardian.

The appointed surveillance officers at the sentinel sites were supervised by DLM staff in order to ensure adequate performance in the detection of cases, data collection and collection and storage of samples. During the supervision visit, knowledge, skills and practices of the surveillance officers related to influenza surveillance procedures were evaluated using a standard evaluation checklist. Ad-hoc trainings were conducted to address any deficiency. These supervisions were carried out monthly at the sentinel sites situated in Kinshasa Province and, due to financial constraints, twice per year at the sites situated in the other provinces. Annual refresher trainings were conducted for all personnel involved in ISS at the participating sites. Individual-level laboratory results were communicated monthly to the sentinel sites and weekly, monthly and quarterly reports were generated and shared among relevant stakeholders. However, no thresholds to assess the intensity and impact of the influenza season were established.

### Evaluation of the influenza surveillance system

We used the CDC guidelines [3, 4] to evaluate the performance of the ISSS in DRC during January 2012 through December 2015. In accordance with the CDC guidelines, the performance of the system was evaluated using eight surveillance attributes: (i) data quality and completeness for key variables, (ii) timeliness, (iii) representativeness, (iv) flexibility, (v) simplicity, (vi) acceptability, (vii) stability and (viii) utility. For each attribute, specific indicators were developed and described using quantitative and/or qualitative methods (Tables 3, 4 and 5). Surveillance databases from syndromic and virological surveillance as well as the laboratory receiving log were analyzed to assess data quality and completeness, timeliness and stability. Furthermore, in order to assess simplicity, acceptability, stability and utility, standardized, pretested and anonymous questionnaires were self-administered by surveillance staff at sentinel sites as well as key informants (i.e., staff involved in ISS or leadership) from the DLM and INRB. All personnel involved in ISS was requested to participate to the questionnaire survey. Three different questionnaires were developed, one for each of the three target groups (i.e. surveillance staff at sentinel sites and staff at the INRB and DLM). Data collected from the surveillance system were also compared with WHO minimum data collection standards for ILI and SARI surveillance [1].

**Table 2:** Mean indicators’ scores (range 1–3) for each attribute used for the evaluation of the influenza sentinel surveillance system in the Democratic Republic of Congo, 2012–2015

Attributes	Number of evaluated indicators	Mean score	Performance
• Data quality and completeness	7	3.0	Good
• Timeliness	4	2.7	Moderate to good
• Representativeness	2	2.0	Moderate
• Flexibility	2	2.0	Moderate
• Simplicity	12	2.9	Moderate to good
• Acceptability	3	2.3	Moderate to good
• Stability	7	2.4	Moderate to good
• Utility	4	2.5	Moderate to good
• Overall	41	2.5	Moderate to good

**Table 3:** List of indicators and scores [1 (< 60%): weak performance; 2 (60–79%): moderate performance; 3 (≥80%) good performance] for data quality and completeness, timeliness, representativeness and flexibility used for the evaluation of the influenza sentinel surveillance system in the Democratic Republic of Congo, 2012–2015

Indicator	Calculation/data inputs	Data source	Indicator value	Score
Data quality and completeness				
• Proportion of SARI/ILI cases that meet the case definition	Number of ILI/SARI cases that meet the case definition / Total number of ILI/SARI cases	Case-based database	97.4%	3
• Proportion of samples from ILI/SARI cases received with accompanying CIF	Number of samples from SARI/ILI cases received with accompanying CIF / Total number of samples received from SARI/ILI cases	Laboratory log book and case-based database	98.0%	3
• Proportion of forms without at least one inconsistent or missing value for key variables^a^	Number of forms without at least one abnormal or missing value / Total number of forms	Case-based database	97.5%	3
• Proportion of good quality samples received	Number of good quality samples received / Total number of samples received	Case-based database	97.0%	3
• Proportion of sample with positive RNP results	Number of samples with a positive RNP result / Total number of samples tested	Case-based database	90.0%	3
• Proportion of sampled ILI/SARI cases with available laboratory results	Number of ILI/SARI cases with available laboratory results / Number of sampled ILI/SARI cases	Case-based database	99.0%	3
• Proportion of collected variables included in the WHO recommended minimum data collection standard	Number of collected variables within the WHO list / Number of WHO recommended variables.	CIF and WHO guidelines for influenza sentinel surveillance.	80.8%	3
Timeliness				
• Proportion of SMS sent on time	Number of SMS sent on time / Number of SMS sent	Aggregated data database	80.0%	3
• Proportion of samples received within 72 h from collection	Number of samples received within 72 h from collection / Number of samples received	Case-based database	96.3%	3
• Proportion of samples tested within one week from receipt	Number of samples tested within one week from receipt / Number of samples tested	Case-based database	90.0%	3
• Proportion of weekly reports issued within five days after the end of the reporting period	Number of weekly reports issued within five days after the end of the reporting period / Number of weekly reports issued	Weekly reports audit	75.0%	2
Representativeness				
• Geographical coverage	Number of provinces covered by the influenza sentinel surveillance network / Total number of provinces	Geographic distribution of sentinel sites.	45.5%	1
• Inclusion of all age groups	Age distribution of cases SARI/ILI (median, minimum and maximum)	Case based database	Med.: 15 Y Min.: 0 Y Max.: 89 Y	3
Flexibility				
• Proportion of samples tested for pathogens other than influenza	Number of samples tested for pathogens other than influenza / Number of samples tested for influenza	Case-based database	37.3%	1
• Number of syndromes surveyed with the influenza surveillance system	Total, respiratory, gastro-intestinal and malaria admissions/consultations reported in the aggregated data form	Aggregated data database	Qualitative assessment	3

Abbreviations: *ILI* influenza-like-illness, *SARI* severe acute respiratory illness, *CIF* case investigation form, *RNP* RiboNucleic Protein, *WHO* World Health Organization, *SMS* Short Message Service

^a^ Key variables evaluated for completeness and consistency of data collection forms: site, age/date of birth, sex, date of consultation admission, date of symptoms onset, date of sample collection and signs and symptoms included in the case definitions

**Table 4:** List of indicators and scores [1 (< 60%): weak performance; 2 (60–79%): moderate performance; 3 (≥80%) good performance] for simplicity used for the evaluation of the influenza sentinel surveillance system in the Democratic Republic of Congo, 2012–2015

Indicator	Calculation/data inputs	Data source	Indicator value^a^	Score
Simplicity				
• Perception of surveillance staff on identification of cases^b^	Number of surveillance staff within each reported category / Number of surveillance staff interviewed	Questionnaire survey among surveillance staff at sentinel sites	VD: 0.0% D: 0.0% E: 82.9% VE: 17.1%	3
• Perception of surveillance staff on obtaining consent^b^	Number of surveillance staff within each reported category / Number of surveillance staff interviewed	Questionnaire survey among surveillance staff at sentinel sites	VD: 0.0% D: 8.6% E: 71.4% VE: 20.0%	3
• Perception of surveillance staff on filling the CIF^b^	Number of surveillance staff within each reported category / Number of surveillance staff interviewed	Questionnaire survey among surveillance staff at sentinel sites	VD: 0.0% D: 2.9% E: 80.0% VE: 17.1%	3
• Perception of surveillance staff on sample collection^b^	Number of surveillance staff within each reported category / Number of surveillance staff interviewed	Questionnaire survey among surveillance staff at sentinel sites	VD: 0.0% D: 5.7% E: 80.0% VE: 14.3%	3
• Perception of surveillance staff on sample collection^b^	Number of surveillance staff within each reported category / Number of surveillance staff interviewed	Questionnaire survey among surveillance staff at sentinel sites	VD: 0.0% D: 0.0% E: 79.4% VE: 20.6%	3
• Perception of surveillance staff on packaging and storage of samples^b^	Number of surveillance staff within each reported category / Number of surveillance staff interviewed	Questionnaire survey among surveillance staff at sentinel sites	VD: 0.0% D: 0.0% E: 82.4% VE: 17.6%	3
• Perception of surveillance staff on completing the screening/enrollment logbook^b^	Number of surveillance staff within each reported category / Number of surveillance staff interviewed	Questionnaire survey among surveillance staff at sentinel sites	VD: 0.0% D: 0.0% E: 82.4% VE: 17.6%	3
• Perception of surveillance staff on sending weekly SMS of aggregated data^b^	Number of surveillance staff within each reported category / Number of surveillance staff interviewed	Questionnaire survey among surveillance staff at sentinel sites	VD: 0.0% D: 6.3% E: 71.9% VE: 21.9%	3
• Time to enroll a SARI/ILI case from the identification to the sample packaging^c^	Number of surveillance staff within each reported category (< 30 min, 30–60 min, > 60 min) / Number of surveillance staff interviewed	Questionnaire for surveillance staff at sentinel sites	< 30: 48.6% 30–60: 40.0% > 60: 11.4%	2
• Perception of INRB laboratory staff on completing the laboratory register^c^	Number of lab staff within each reported category / Number of laboratory staff interviewed	Questionnaire survey among laboratory staff at INRB	VD: 0.0% D: 0.0% E: 66.7% VE: 33.3%	3
• Perception of INRB laboratory staff to implement testing procedures^c^	Number of lab staff within each reported category / Number of laboratory staff interviewed	Questionnaire survey among laboratory staff at INRB	VD: 0.0% D: 0.0% E: 100.0% VE: 0.0%	3
• Time to implement all steps of analysis of laboratory testing^b^	Number of laboratory staff within each reported category (< 30 min, 30–60 min, > 60 min) / Number of laboratory staff interviewed	Questionnaire survey among laboratory staff at INRB	< 30: 0.0% 30–60: 100.0% > 60: 0.0	3

Abbreviations: *ILI* influenza-like-illness, *SARI* severe acute respiratory illness, *SMS* short message service, *INRB* Institut National de Recherche Biomédicale, *CIF* Case Investigation Form

^a^ VD: very difficult; D: difficult; E: easy; VE: very easy. The combined percentage of “easy” and “very easy” was used to obtain the score

^b^ 35 surveillance staff at the sentinel sites out of 39 targeted responded to the questionnaire survey

^c^ 3 laboratory scientists at the INRB out of 4 targeted responded to the questionnaire survey

For consistency and comparability of findings we used the evaluation method and scoring system utilized for influenza surveillance evaluations conducted in other African countries [7–10]. A scale from 1 to 3 was used to provide a score for each quantitative indicator as follows: < 60% scored 1 (weak performance); 60–79% scored 2 (moderate performance); ≥80% scored 3 (good performance) [8]. For qualitative indicators a score was assigned based on the same scale using expert consensus. Thereafter the scores assigned to each indicator were averaged for all indicators evaluated within each attribute to provide an overall score for each surveillance attribute assessed in this study. An overall score for the surveillance system was obtained by averaging the eight mean attribute scores. This evaluation was implemented by personnel from the KSPH (internal evaluators) and CDC (external evaluators) and was not linked to the Joined External Evaluation conducted in DRC in 2018.

### Data analysis

Categorical variables were expressed as percentage of outcomes of interest over total observations for each quantitative indicator. Furthermore, 95% confidence intervals for proportions were calculated using the binomial distribution. The statistical analysis was implemented using Stata version 14.2 (StataCorp, College Station, Texas, USA).

## Results

### Implementation of sentinel surveillance and questionnaire survey

**Fig. 2:**
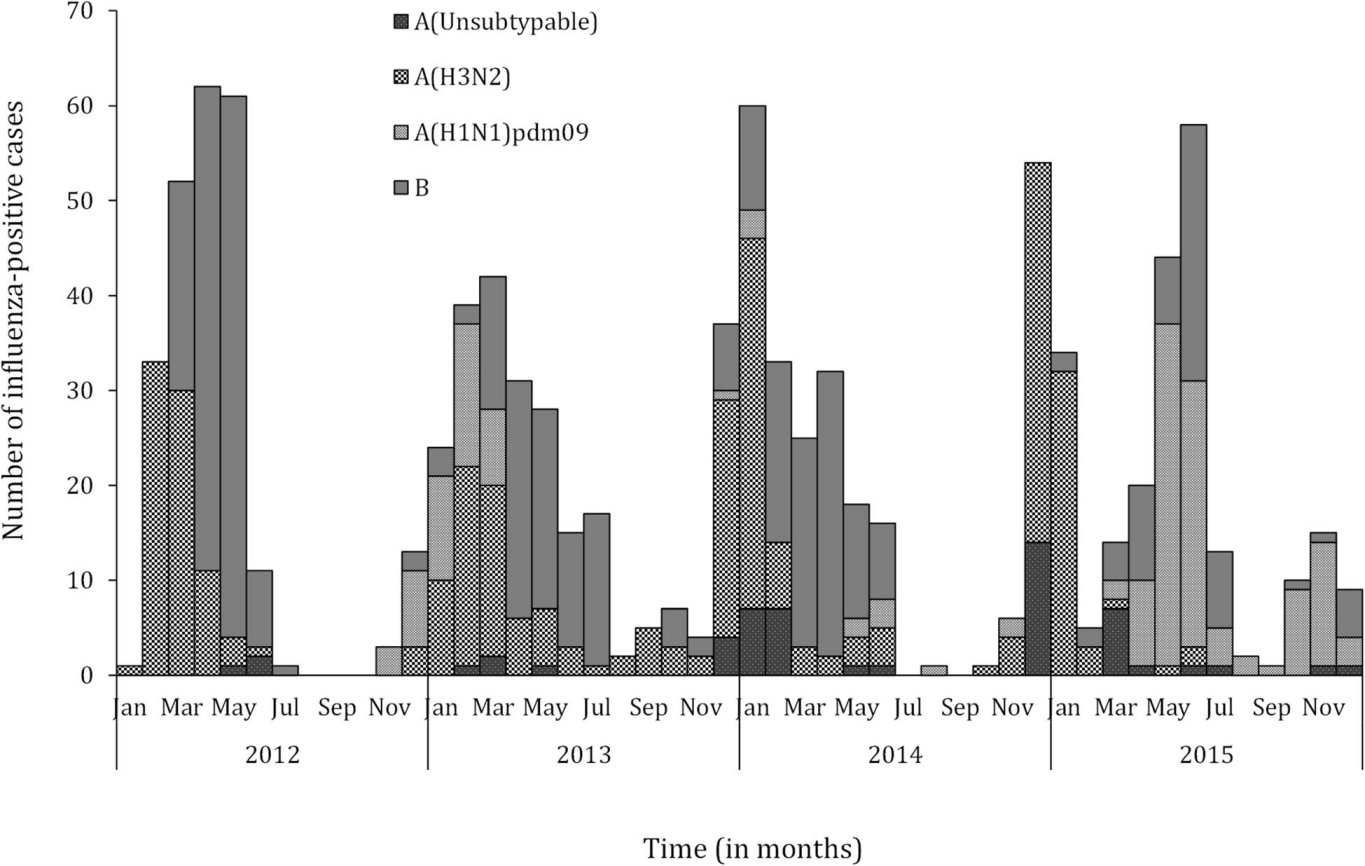
Monthly number of influenza-positive specimens among patients with influenza-like illness or severe acute respiratory illness from 11 surveillance sites, Democratic Republic of Congo, 2012–2015

During 2012–2015, 16,152 patients with respiratory illness were reported from the 11 sentinel surveillance sites. Of these, 11,737 (72.7%) were outpatient consultations and 4415 (23.3%) were hospital admissions of which 4812 (40.9%) and 2869 (64.9%) met the ILI and SARI case definitions, respectively. Of the 7690 patients eligible for enrollment, 7090 (92.2%) were enrolled in the surveillance system; 4339/4821 (90.0%) and 2751/2869 (95.9%) among patients with ILI and SARI, respectively. Influenza viruses were detected in 597/7090 (8.4%; 95% CI: 7.8–9.1%) samples tested. Of these, 111 (18.6%) were influenza A(H1N1)pdm09 viruses, 209 (35.0%) were influenza A(H3N2) viruses, 27 (4.5%) were influenza A viruses not subtyped and 251 (42.0%) were influenza B viruses (Fig. 1). Influenza viruses were detected in 446/4339 (10.3%; 95% CI: 9.4–11.2%) samples from patients with ILI and in 151/2751 (5.5%; 4.7–6.4%) samples from patients with SARI. Influenza viruses were detected mainly during December to May (Fig. 2). The questionnaire survey was completed by 35/39 (89.7%), 3/4 (75.0%) and 6/6 (100.0%) personnel involved in influenza surveillance at sentinel sites, INRB and DLM, respectively.

### Evaluation of the surveillance system

The overall mean score for the ISSS in DRC was 2.5 (moderate to good performance out of a 1–3 scale) (Table 2).

#### Data quality and completeness

All seven evaluated indicators had good performance (Table 3). Of the seven evaluated indicators, the proportion of collected variables included in the WHO minimum data collection standard scored the lowest (80.8%). Information about the use of antivirals and the presence of some underlying medical conditions were not collected in the patient’s CIF. The mean score for data quality and completeness was 3.0 (good performance) (Table 2).

#### Timeliness

Of the four indicators evaluated three had good performance and one had moderate performance (Table 3). Delays in issuing the weekly surveillance reports within 5 days after the reporting period were observed in 25% of instances. The mean score for timeliness was 2.7 (moderate to good performance) (Table 2).

#### Representativeness

Of the 2 indicators evaluated 1 had good performance and 1 (geographic representativeness) had weak performance (Table 3). Sentinel sites were situated only in 5 of 11 (45.5%) provinces. The mean score for representativeness was 2.0 (moderate performance) (Table 2).

#### Flexibility

Of the two indicators evaluated, one had good performance and one had weak performance (Table 3). Whereas, the ISSS demonstrated flexibility in monitoring also non-respiratory syndromes (i.e. gastro-intestinal and malaria as well as total admissions or consultations), the proportion of samples tested for other pathogens was low (37.3%) and implemented only during a short period because of limited funds. The mean score for flexibility was 2.0 (moderate performance) (Table 2).

#### Simplicity

Of the 12 indicators evaluated, 11 had good performance and one had moderate performance (Table 4). All of the eight indicators used to assess the perception of surveillance personnel at sentinel sites to implement different surveillance activities had good performance. Nevertheless, the surveillance procedures from the identification of cases to the final packaging of samples took > 30 min for 51.4% (18/35) of staff. All three indicators used to assess the perception of laboratory personnel to conduct influenza diagnostic testing had good performance. The mean score for simplicity was 2.9 (moderate to good performance) (Table 2).

#### Acceptability

Of the three indicators evaluated, two had good performance and one had weak performance (Table 5). Staff involved in influenza surveillance were expected to also conduct clinical work for 70% of their time. On average, influenza surveillance activities occupied 48.3% of the time of the surveillance staff. The mean score for acceptability was 2.3 (moderate to good performance) (Table 2).

**Table 5:** List of indicators and scores [1 (< 60%): weak performance; 2 (60–79%): moderate performance; 3 (≥80%) good performance] for acceptability, stability and utility used for the evaluation of the influenza sentinel surveillance system in the Democratic Republic of Congo, 2012–2015

Indicator	Calculation/data inputs	Data source	Indicator value	Score
Acceptability				
• Proportion of surveillance staff that is satisfied with the weekly bulletins^a^	Number of surveillance staff within each reported category [not satisfied (NS), poorly satisfied (PS), satisfied (S), very satisfied (VS)] / Number of surveillance staff interviewed	Questionnaire for surveillance staff at sentinel sites	NS: 0.0% PS: 0.0% S: 30.0% VS: 70.0%	3
• Proportion of surveillance staff that is satisfied with supervision and feedback^a^	Number of surveillance staff within each reported category [not satisfied (NS), poorly satisfied (PS), satisfied (S), very satisfied (VS)] / Number of surveillance staff interviewed	Questionnaire for surveillance staff at sentinel sites	NS: 0.0% PS: 17.6% S: 44.1% VS: 38.2%	3
• Proportion of time allocated to influenza surveillance activities per week^a^	Number of hours allocated to influenza surveillance activities per week / Number of working hour per week	Questionnaire for surveillance staff at sentinel sites	48.3%	1
Stability				
• Frequency of lack of data collection forms^a,c^	Number of surveillance sites within each reported category [never (0), once per year (1), 2–3 times per year (2–3), ≥4 times per year(≥4)] / Number of surveillance sites	Questionnaire for surveillance staff at sentinel sites	0: 100.0% 1: 0.0% 2–3: 0.0% ≥4: 0.0%	3
• Frequency of lack of sampling material^a^	Number of surveillance sites within each reported category [never (0), once per year (1), 2–3 times per year (2–3), ≥4 times per year(≥4)] / Number of surveillance sites	Questionnaire for surveillance staff at sentinel sites	0: 72.7% 1: 27.3% 2–3: 0.0% ≥4: 0.0%	2
• Frequency of lack of credits for SMS^a^	Number of surveillance sites within each reported category [never (0), once per year (1), 2–3 times per year (2–3), ≥4 times per year(≥4)] / Number of surveillance sites	Questionnaire for surveillance staff at sentinel sites	0: 100.0% 1: 0.0% 2–3: 0.0% ≥4: 0.0%	3
• Frequency at which the transport of samples was delayed^a^	Number of surveillance sites within each reported category [never (N), seldom (S), often (O), regularly (R)] / Number of surveillance sites	Questionnaire for surveillance staff at sentinel sites	N: 0.0% S: 90.9% O: 9.1% R: 0.0%	2
• Frequency at which the refrigerators of the sentinel sites were not functional^a^	Number of surveillance sites within each reported category [never (N), seldom (S), often (O), regularly (R)] / Number of surveillance sites	Questionnaire for surveillance staff at sentinel sites	N: 100.0% S: 0.0% O: 0.0% R: 0.0%	3
• Frequency at which a power failure, including the generator, occurred at the surveillance sites^a^	Number of surveillance sites within each reported category [never (N), seldom (S), often (O), regularly (R)] / Number of surveillance sites	Questionnaire for surveillance staff at sentinel sites	N: 0.0% S: 9.1% O: 18.2% R: 72.7%	1
• Proportion of sentinel sites with at least one member of staff trained in sentinel surveillance procedures during the last one year^a^	Number of sentinel sites with at least one trained member of staff / Number of surveillance sites	Questionnaire for surveillance staff at sentinel sites	100.0%	3
Utility				
• Number of decisions taken by the INRB and/or the DLM based on influenza sentinel surveillance results^b,d^	N/A	Questionnaire survey for DLM and INRB	4	2
• Proportion of surveillance staff that receive the following reports: (i) Virological surveillance report, (ii) Syndromic surveillance report, (iii) Influenza bulletin^a^	Number of surveillance staff that receive reports / Number of surveillance staff	Questionnaire for surveillance staff at sentinel sites	77.1%	2
• Estimation of burden of influenza-associated illness using surveillance data	Not applicable	Publication on burden of influenza-associated ILI and SARI.	1 [13]	3
• Contribution to influenza Regional/Global studies	Not applicable	Publications on Regional/Global studies with DRC influenza data	3 [14, 15]	3

Abbreviations: *ILI* influenza-like-illness, *SARI* severe acute respiratory illness, *SMS* short message service, *INRB* Institut National de Recherche Biomédicale, *DLM* Direction de la Lutte contre les Maladies

a 35 surveillance staff at the sentinel sites out of 39 targeted responded to the questionnaire survey

b 3 laboratory scientists at the INRB out of 4 targeted and 6 staff at the DLM out of 6 targeted responded to the questionnaire survey

c No information on the duration of lack of surveillance material was collected

d Decisions taken in relation to the data generated from the ISSS: (i) investigation of respiratory outbreaks in Kinshasa in 2013; (ii) formulation of outbreak investigation and response guideline for influenza outbreaks; and (iii) inclusion of influenza virus in the list of epidemic-prone notifiable diseases

#### Stability

Of the seven indicators evaluated, four had good performance, two had moderate performance and one had poor performance (Table 5). The main aspects that affected stability were elevated frequencies of electricity cuts and generator failures, delays in sample transportation and occasional lack of sampling material in some remote sentinel sites. In addition, the ISSS in DRC is mainly funded (> 90%) by international agencies. The mean score for stability was 2.4 (moderate to good performance) (Table 2).

#### Utility

Of the four indicators evaluated, two had good performance and two had moderate performance (Table 5). The mean score for utility was 2.5 (moderate to good performance) (Table 2). In addition to the measured indicators, respondents from the sentinel sites, DLM and INRB reported that the ISSS contributed to: (i) a better understanding of the epidemiology, circulating patterns and proportional contribution of influenza virus among patients with ILI or SARI; (ii) acquisition of new key competences related to surveillance of respiratory pathogens, including identification of cases and laboratory diagnosis; and (iii) continuous education of surveillance staff and clinicians at sentinel sites about influenza and other respiratory pathogens. However, due to limited resources no actions were undertaken to mitigate the impact of seasonal influenza epidemics.

## Discussion

During 2012–2015, the ISSS in DRC performed well with an overall system score of 2.5 (moderate to good performance) on a 3-point scale. In line with its objectives, the utility of the system was demonstrated by its ability to monitor the circulating influenza viruses in the country, monitor the temporal trends of influenza virus circulation, assess the proportional contribution of influenza-associated illness among outpatients and inpatients with ILI or SARI [6], estimate the national burden of influenza-associated illness [13] and contribute to the regional and global understanding of influenza epidemiology, including sharing of clinical samples with WHO collaborating center for annual selection of vaccine strains [5, 14, 15].

The flexibility of the ISSS in DRC allowed monitoring several syndromes of importance for the country under the same platform, increasing cost-effectiveness and avoiding the implementation of vertical surveillance programs. The flexibility of the system was demonstrated also for laboratory-based surveillance that, for instance, was able to monitor the circulation of several respiratory pathogens in the country; however, testing for pathogens other than influenza was not systematic because of lack of resources.

A strength of the system was the selection of the sentinel sites, which allowed the implementation of both ILI and SARI surveillance in most of the selected facilities, reducing specimens transport and supervision cost. Whereas the surveillance system was implemented in five of six target provinces, six of 11 provinces of the country were not covered. Adding additional surveillance sites could improve representativeness; however, this would also increase costs and logistical difficulties. Given that the existing system met the surveillance objectives (especially those related to seasonal influenza), adding surveillance sites in other provinces is not recommended. The identification of avian influenza viruses’ infection in humans (one of the objectives of the surveillance system) through facility-based sentinel surveillance remains challenging due to the limited number of surveillance sites that can be supported with limited resources. Close collaboration with the animal health authorities and the joint investigation of animal and human populations during identified avian influenza outbreaks in birds would represent a more cost-effective strategy to identify potential zoonotic transmission of avian influenza viruses.

System stability was demonstrated by its ability to operate continuously since its establishment in 2006. The stability of most surveillance systems in the African region is related to human and financial resources. The stability of the ISSS in DRC is probably reliant on its simplicity that resulted in the acceptability of surveillance activities by surveillance staff. Nonetheless, the simplicity of the system may have affected the extent of information collected. Not all minimum data collection requirements suggested by WHO for influenza sentinel surveillance [1] were met. The collection of additional data about underlying medical conditions may allow, for instance, the identification of more vulnerable populations for severe influenza-associated illness that in return could guide targeted prevention measures such as annual influenza immunization [16, 17]. Nevertheless, the introduction of more intense data gathering should be weighed against its impact on acceptability. Whereas the system overall was stable, logistical constrains in terms of power supply and transport of samples were identified. The simplicity and acceptability of the system as well as the periodic supervision of the sentinel sites may also have contributed to the observed good quality and completeness of the data. Nonetheless, the ISSS in DRC is largely reliant on external funds (> 90%) and the acceptability of maintaining the surveillance system through national funds should be evaluated and/or contemplated as lack of external funds could impact the stability of the system.

The use of an SMS-based system for the transmission of weekly aggregated data, verified thereafter by the use of data collection forms, were key components that contributed to both data quality and completeness as well as timeliness as observed in other settings [8]. While the SMS-based system allowed the timely transmission of information even from remote sites, some delays were experienced in the shipment of samples or restocking of surveillance material. Logistical challenges such as those are common in the African region and are expected when a geographically representative coverage is attempted. Overall, the timeliness of the system in relation to its geographical coverage is considered satisfactory.

In conclusion, the ISSS in DRC performed satisfactorily and provided reliable and timely data on the circulation of influenza viruses in the country. The simplicity and acceptability of the system are key factors that contributed to its stability. The ISSS currently relies on 11 sentinel sites. Given the dependency of the system on external funds, the system could be decreased in size to allow continuation of implementation with local funds in the future. The collection of additional information on underlying medical conditions may enable the continuous monitoring of groups at increased risk for severe influenza. These data would also align the DRC system with the minimum data collection requirements suggested by WHO [1].

## Conclusions

The system performed overall satisfactorily and provided reliable and timely data about influenza circulation in DRC. The simplicity of the system contributed to its stability. While the surveillance system is stable and able to identify circulating influenza strains, the data being generated is not fully utilized as DRC lacks guidelines on the use of antivirals and vaccines as well as non-pharmaceutical interventions for influenza. A better use of the available data could be made to inform and promote prevention interventions especially among the most vulnerable groups.

## Abbreviations

CDC: Centers for disease control and prevention
CIF: Case investigation form
DLM: Direction de la lutte contre les maladies
DRC: Democratic Republic of Congo
GISRN: Global influenza and response network
ILI: Influenza-like illness
INRB: Institut national de recherche biomédicale
ISSS: Influenza sentinel surveillance system
KSPH: Kinshasa school of public health
MoH: Ministry of health
SARI: Severe acute respiratory illness
SMS: Short message service
URT: Upper respiratory tract
WHO: World health organization
